# STATdb: A Specialised Resource for the STATome

**DOI:** 10.1371/journal.pone.0104597

**Published:** 2014-08-26

**Authors:** C. Pawan K. Patro, Asif M. Khan, Tin Wee Tan, Xin-Yuan Fu

**Affiliations:** 1 Department of Biochemistry, Yong Loo Lin School of Medicine, National University of Singapore, Singapore, Singapore; 2 Department of Pharmacology and Molecular Sciences, Johns Hopkins University School of Medicine, Baltimore, Maryland, United States of America; 3 Perdana University Graduate School of Medicine, Serdang, Selangor Darul Ehsan, Malaysia; 4 Cancer Science Institute of Singapore (CSI), National University of Singapore, Singapore, Singapore; 5 Department of Microbiology and Immunology, Indiana University School of Medicine, Indianapolis, Indiana, United States of America; University of Regensburg, Germany

## Abstract

Signal transducers and activators of transcription (STAT) proteins are key signalling molecules in metazoans, implicated in various cellular processes. Increased research in the field has resulted in the accumulation of STAT sequence and structure data, which are scattered across various public databases, missing extensive functional annotations, and prone to effort redundancy because of the dearth of community sharing. Therefore, there is a need to integrate the existing sequence, structure and functional data into a central repository, one that is enriched with annotations and provides a platform for community contributions. Herein, we present STATdb (publicly available at http://statdb.bic.nus.edu.sg/), the first integrated resource for STAT sequences comprising 1540 records representing the known STATome, enriched with existing structural and functional information from various databases and literature and including manual annotations. STATdb provides advanced features for data visualization, analysis and prediction, and community contributions. A key feature is a meta-predictor to characterise STAT sequences based on a novel classification that integrates STAT domain architecture, lineage and function. A curation policy workflow has been devised for regulated and structured community contributions, with an update policy for the seamless integration of new data and annotations.

## Introduction

Signal transducers and activators of transcription (STAT) proteins are one of the most important signalling molecules in metazoans [1], [2], [3], playing dual roles as cytoplasmic signalling proteins and nuclear transcription factors in the cell. STATs are key components of the Janus Kinase (JAK)/STAT signalling pathway [4], an evolutionarily conserved cascade that facilitates a wide range of inter- and intra-cellular signalling roles vital for cellular differentiation, growth and survival [5], [6], [7]. STATs get activated via phosphorylation by kinases, such as JAKs and Src kinases, and growth factor receptors, among other activating proteins, responding to extracellular-signalling proteins [8], [9]. STAT proteins, upon activation, translocate to the nucleus to regulate a diverse set of target genes [1], however several deviations to this canonical pathway have been described to date [10]. Numerous studies have shown that dysregulation of the JAK/STAT pathway is associated with chronic inflammation, neurodegenerative diseases and cancer, among other disease states [11].

The STAT protein family in mammals comprises seven members—STAT1-4, STAT5A and 5B, and STAT6—with diverse functions [1], [11], [12]. Knockout of either STAT1 or STAT2 results in an impaired response to interferons [1]. Furthermore, the absence of STAT1 results in impaired growth control [13] whereas STAT2 knockout mice show numerous defects in their immune response [14]. Early embryonic lethality has been associated with STAT3 knockout mice [1], [13], and additional complications, such as multiple defects in adult tissues and an impaired response to pathogens, are also linked to the absence of STAT3. STAT4 deletion affects T helper 1 (TH1) cell function, opposing STAT6 function, which impairs TH2 differentiation [1], [13]. Both STAT5A and STAT5B are important for breast development/lactation: STAT5A is required for prolactin responsiveness, whereas STAT5B is required for growth hormone responsiveness [1], [13]. STAT5 refers to the gene that duplicated to give rise to STAT5A and STAT5B in species ancestral to mammals [15]. Both STAT5.1 and STAT5.2 are STAT5 homologs in fishes [15].

STAT family of proteins has thus been studied intensively [1], [11], which has led to the accumulation of sequence and structure data scattered across various public databases. For example, the primary NCBI sequence databases (GenBank and GenPept) are comprehensive but lack extensive functional annotations, such as status of experimental validation, STAT domains, interacting proteins, and gene and structural information, which are found in other databases, such as UniProt, RefSeq, PDB, Gene, CDD, and within the literature. Public databases, however, are prone to errors [16], and consequently an extensive analysis is required to ascertain the reliability of data in public domains by cross-checking with other databases and with what is cited in the literature. This difficult task, along with the substantial lack of sharing amongst the scientific community, has thus led to redundant efforts in the laboratory. Therefore, there is an urgent need to assemble, organize, remove duplicates and integrate existing sequence, structure and functional data into a central repository that is enriched with annotations and provides a platform for community contribution to allow for systematic, integrated analyses of STATs.

Herein, we present STATdb, a specialised repository of STAT sequences, representing the known STATome, integrating existing sequence, structure and functional information from various databases, and the literature, and including manual annotations. This, to our knowledge, is the first reported specialised Web resource for STAT sequences. STATdb, besides the basic functionalities such as database query using keyword search and data download, provides advanced features for data visualization, analysis and prediction, and community contribution. Users can dynamically browse the STATome—the complete dataset of reported STAT sequence records in STATdb—and interactively view available 3D structures. STATdb is integrated with sequence analysis tools, such as the Basic Local Alignment Search Tool (BLAST) for sequence similarity searches and ClustalW for multiple sequence alignments on the fly. A key feature of the database is STATdbPredict, which is used to characterize STAT sequences based on a novel classification scheme that incorporates domain architecture, lineage and function. Sequence records are manually annotated with STATdb classification notation, experimental status validation, and individual domain sequences, among others. A submission/curation policy workflow has been devised for regulated and structured contribution of new records and for enrichment/correction of functional annotations of existing records by the STAT research community (curator) through an easy-to-use interface. Community contribution, based on existing data and literature, is important in biological data-warehousing [17] and the approach has been highly successful, as exemplified by numerous Wiki-based projects: PDBwiki [18], WikiProteins [19], Gene Wiki [20], RNA Wikiproject [21], EcoliWiki [22] and WikiPathways [23]. Additionally, an update policy has been devised for the regular integration of new records and annotations from public databases and/or the community.

## Materials and Methods

### Sequence Data Collection

Protein and nucleotide sequences of STAT were first collected through keyword searches using the National Center for Biotechnology Information (NCBI) Reference Sequence (RefSeq) [24], [25] database, followed by sequence similarity searches against all reported sequences in the NCBI non-redundant (NR) database [25]. Keyword hits were manually checked and verified as STAT according to the literature. Selected verified sequences were used as query for Position-Specific Iterated (PSI)-BLAST search [26] in order to perform a comprehensive survey of STAT sequences. Significant blast hits were selected and sequence duplicates were removed using CD-HIT [27]. The remaining non-redundant sequences were used to populate STATdb.

### Database Record Annotation

Existing STAT record annotations in various public databases were studied to identify relevant fields for STATdb. The list of fields defined for STATdb records are provided in Table 1. Fields that provide information selected from the source record (NCBI Entrez Protein Database) are marked as “Source”, such as gene name, protein name, type of STAT, database cross-references, literature, species, location of the gene on the chromosome, length of the protein sequence, and amino acid sequence for the full-length protein, as well as the list of individual STAT domains. “Assigned” fields are those not found in the source record, but were included to provide information obtained from database cross-references and/or analysis of the sequence data, existing annotations or the literature.

**Table 1:** List of all fields defined for STATdb records.

Field Name	Description	Source#/Assigned
STATdb Id	STATdb Unique Identifier/Accession Number	**Assigned** (by STATdb authors)
gName	Gene Name	Source & **Assigned** (via NCBI Entrez Gene database)
pName	Protein Name	Source
STAT type	STAT family sub-group based on function	Source & **Assigned** (literature$)
STATdb Classification	Classification based on three-tier system	**Assigned** (by STATdb authors)
	Domain Architecture - Lineage - Function	
DBXRef	Database Cross References	Source & **Assigned** (pathway information obtained via KEGG database and other cross references are from source)
Literature	Literature (PubMed Reference Id)	Source
Species (Source Organism)	Species containing STAT	Source
Expt. Status	Experimental Status	**Assigned** (by STATdb authors)
	E - Experimentally Verified	
	P - Predicted/Hypothetical	
	U - Unknown	
Expt. Status Evidence	Experimental Status Evidence	**Assigned** (literature$)
ChromLoc	Chromosome Location	Source
IntPartners	Interacting Proteins	**Assigned** (via NCBI Entrez Gene database)
SeqLen	Sequence Length (Protein)	Source
Completeness	Completeness of the protein sequence	**Assigned** (by STATdb authors)
	Complete/Incomplete	
STAT Dom	STAT domains	Source
DomArchitecture	Domain Architecture	**Assigned** (via SMART database)
STAT DomSeq	Nucleotide & Protein Sequence of STAT domains	**Assigned** (derived from source)
STAT_int	Nucleotide & Protein Sequence for protein interaction domain	**Assigned** (derived from source)
STAT_alpha	Nucleotide & Protein Sequence for all alpha domain	**Assigned** (derived from source)
STAT_bind	Nucleotide & Protein Sequence for DNA binding domain	**Assigned** (derived from source)
STAT_sh2	Nucleotide & Protein Sequence for SH2 domain	**Assigned** (derived from source)
STAT_taz2	Nucleotide & Protein Sequence for TAZ2 domain	**Assigned** (derived from source)
BindingMotif	DNA Binding Motif	**Assigned** (via JASPAR database)
NucSeq	Nucleotide Sequence	**Assigned** (via NCBI Entrez Nucleotide database)
ProtSeq	Protein Sequence	Source
Comment	STATdb Curation Comments	**Assignable**

Fields that provide information selected from the source record (NCBI Entrez Protein database) are marked as “Source”. “Assigned” fields are those not found in the source record, but were included to provide information obtained from analysis of the sequence data, existing annotations or the literature.

# *NCBI Entrez protein database.*

$ *The respective literature are indicated in the relevant records.*

### STATdb Classification

STATdb-enriched annotations enabled the construction of a novel classification scheme for the characterization of STAT sequences and for the prediction of novel family members. This classification is based on a three-tier system: “Domain Architecture – Lineage – Function”.

“Domain Architecture”, or “DA”, is used to describe the observed order/arrangement of STAT domains within the protein. STAT proteins comprise five major domains: protein interaction domain (STAT_int), all-alpha domain (STAT_alpha), DNA-binding domain (STAT_bind), SH2 domain (SH2) and the transactivation domain (TAZ2). The five unique domain architectures are referred to as DA I – DA V, observed to date for STATdb sequences with DA U representing uncommon combinations and artificial sequences.

“Lineage” is defined as a sub-classification of “Domain Architecture”, and is based on the taxonomy of the species from which the STAT sequence was isolated. All STATdb sequences of each domain architecture were analysed for their species lineage by interrogating the NCBI Taxonomy Database and the sequences were then grouped according to the furthest common differentiation level from the root (*i.e.*, cellular organism). As STATs are a family of paralogous loci (*e.g.*, in vertebrates), the classification does not aim to coincide the species and gene trees in instances where it is not possible.

“Function” is defined as a sub-classification of “Lineage”, and is based on the role of the STAT family members. Although there are seven mammalian STATs (STAT1-4, STAT5A, STAT5B and STAT6), numerous other STATs are commonly found in fishes or invertebrates, and the functions of these other STATs are also incorporated in this sub-classification tier.

### STATdbPredict

STATdbPredict is a meta-prediction system designed to characterize protein sequences based on STATdb classification. Users can submit one or more sequences in FASTA format to obtain a prediction of DA, lineage and/or function. The prediction process involves querying for the presence of statistically reliable STAT domains using Reversed Position Specific (RPS)-BLAST [26] and classifying them based on “DA”; this is followed by identifying the highest scoring pair (HSP) for the prediction of “Lineage” (tier two) and “Function” (tier three) using BLASTp [26] (see “STATdb Home > Help > Tools: Predict” for the prediction algorithm of STATdbPredict). STAT domain Position Specific Scoring Matrices (PSSMs) were downloaded from the NCBI conserved domain database (CDD) and used to create a local in-house RPS-BLAST–searchable database. This in-house searchable database is of much smaller size than the original CDD; thus, the values of the search output parameters (E-value, percentage identity, alignment length and bit score) will not be the same between the in-house database and original CDD. Since the E-value appears to be inversely proportional to database size [26], its values are larger for the in-house database and, thus, are deemed not appropriate as a parameter for the selection of significant domain hits. Therefore, the experimentally verified STAT records were used to determine the acceptable value range for the remaining other vital parameters (percentage identity, alignment length and bit score) for each domain. The minimum range values of the three parameters (percentage identity, alignment length, and bit score) are used as a cut-off for statistical reliability of a domain hit (see “STATdb Home > Help > Tools: Predict” for the range values). The HSP is used to ascribe “Lineage” and “Function”, and is defined as the best match to the query, with a percentage sequence identity of ≥90 and a length difference of ≤10; predictions based on HSPs that do not meet these criteria are indicated as hits of low confidence.

The accuracy of the prediction system was tested using a test dataset comprising new STAT sequences (as at June 2013) not found in STATdb (as at April 2013, STATdb comprised 1,424 records). These new sequences were obtained using the PSI-BLAST search against the NCBI NR database. The search resulted in 116 new STAT sequences, of which 20 were assigned a “DA U”. The remaining 96 classifiable sequences were non-redundant and used as positive samples for the test dataset, with the top 96 non-redundant, non-STAT hits from the PSI-BLAST used as negative samples. After this analysis was complete, the 116 new STAT sequences were added to STATdb.

### STATdb Construction

STATdb was created using MySQL (www.mysql.com) and the user interface was developed through the use of PHP, HTML and jQuery. MySQL is used for data storage, processing and retrieval of specific information. PHP (www.php.net) pages are used to process the forms and browse through the different sections of the database. HTML was utilised for the website design, with dynamic record browsing according to different groupings facilitated by jQuery (www.jquery.com), which is used to manage all the Java Scripts and AJAX. Analysis tools supported by BioSLAX (www.bioslax.com), such as BLAST similarity search and ClustalW [28] for multiple sequence alignments, are included in the database.

## Results

### Features of STATdb

**Figure 1:**
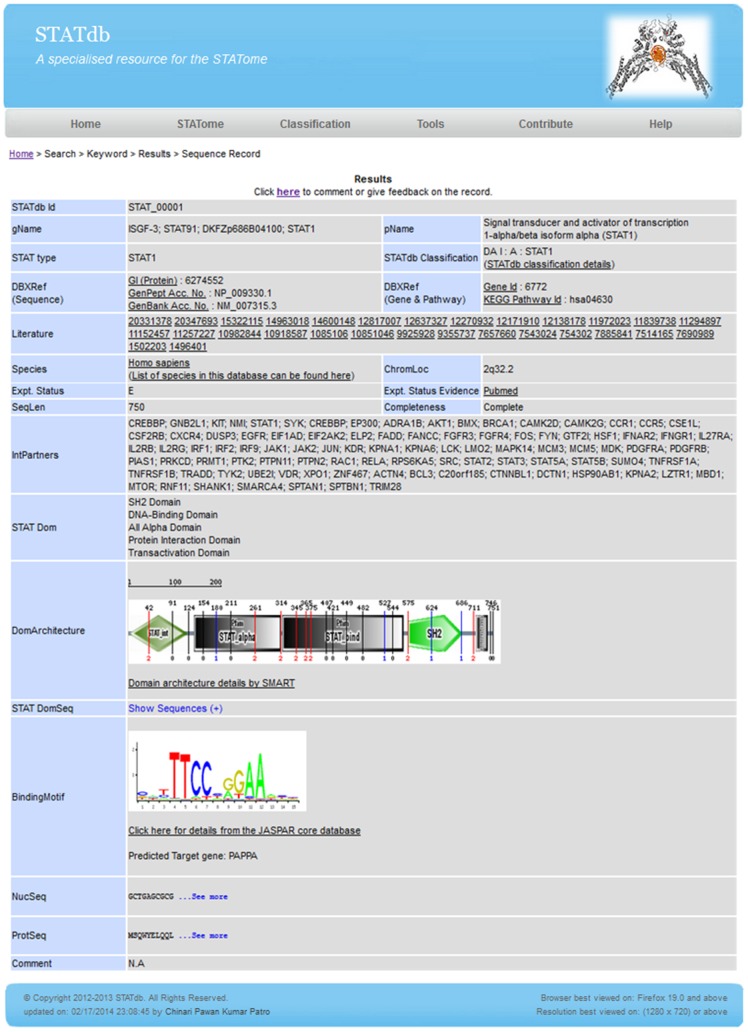
A sample STATdb record.

Each record in STATdb is given a unique Id in the form of “STAT_XXXXX”, where “XXXXX” represents five numerical digits. A sample record is provided in Figure 1. The records comprise standard data fields from the source databases (NCBI Entrez Protein database) and “Assigned” fields, which are defined by the authors for enriched manual annotations:

- STATdb Id – provides an Id for each individual STATdb record.
- Gene name – provides the gene name obtained from the NCBI Entrez Gene database.
- STAT type – STAT family sub-group.
- STATdb classification – provides a notation that earmarks the characteristic features of the sequence in terms of “DA”, “Lineage” and “Function” (see “Classification” section below for details).
- DBXRef – provides database cross-references that are mostly obtained from the source record; however, pathway information is obtained from the KEGG database.
- Experimental status validation – provides information from the literature and/or cross-referenced databases on the reliability of the STAT sequence, as either experimentally verified (E), or hypothetical/predicted (P) or unknown (U).
- IntPartners – lists the interacting protein partners of STAT, which were obtained from NCBI Entrez Gene database.
- Completeness of the protein sequence – the sequence is considered “complete” if all of the domains for the corresponding architecture are present.
- STAT Domain architecture – describes the order of the STAT domains in the sequence (via SMART [29], [30] database).
- STAT Domain sequences – lists the amino acids and the corresponding nucleotide sequences (obtained by use of TBLASTN) of the individual STAT domains.
- Binding Motif – provides the STAT binding motif and the predicted target gene information obtained from JASPAR database.
- NucSeq – provides the nucleotide sequence obtained from the NCBI Entrez Nucleotide database for the corresponding protein.
- STATdb curation comments – this provides a platform for annotations and/or corrections by the STATdb community.

The key features of STATdb can be divided into basic and advanced, as described below:

**A. Basic:**

**i. Keyword and Sequence Search**

Keyword queries of the database include STATdb_Id, gene name, protein name, STAT type, species, STAT domain, interacting proteins or other database cross-references. A sequence search is performed using BLAST against databases of (i) experimentally verified sequences, (ii) predicted, (iii) all STAT sequences (protein and nucleotides) and (iv) interacting partners (JAK, EGFR, and Src Kinase).

**ii. Downloads**

STATdb sequences categorized as “all sequences”, “experimentally verified”, “predicted” and sequences of interacting partners are available for downloaded in FASTA format from the download page.

**B. Advanced:**

**i. Browser**

**Figure 2:**
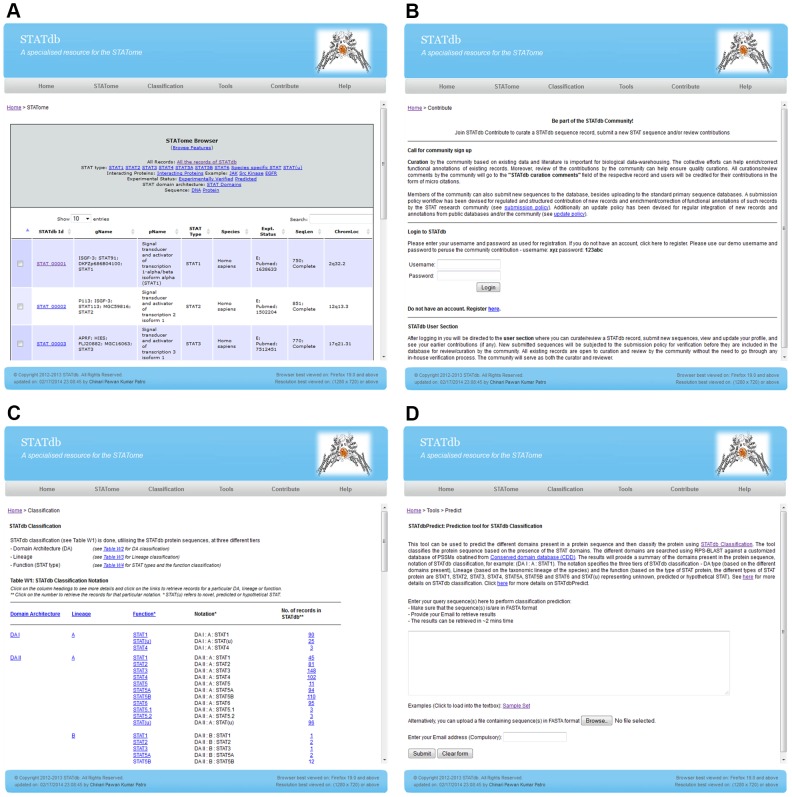
Snapshots of selected STATdb key features. A) STATome Browser – allows for the dynamic browsing of the STATome, a complete set of reported STAT records in STATdb. B) Contribute – provides a platform for the STATdb community to curate annotations or submit new STAT sequences. C) Classification - provides a notation that describes the grouping of a sequence based on our three-tier classification system: “Domain Architecture – Lineage – Function” and D) Predict - characterizes protein sequences using STATdb classification.

The Browser allows for dynamic browsing of the STATome according to all records, types of STAT, DNA or protein sequences, interacting proteins, status of experimental validation, and STAT DA (Figure 2A). Records can be selected to retrieve the full data or only the sequences in FASTA format, or they can be submitted for multiple sequence alignment on the fly using ClustalW.

**ii. View 3D**

The Jmol viewer allows for the manipulation of available 3D structures of STAT obtained from PDB. Currently, there are only 11 reported solved 3D structures for human (2), mouse (8) and the social amoeba *Dictyostelium discoideum* (1). Users can analyse the structures using the different options provided and also download primary sequences (FASTA format) and 3D structure coordinates (PDB format).

**iii. Contribute**

“Contribute” offers a platform for the STATdb community to curate annotations or submit new STAT sequences (Figure 2B). The submission of new STAT sequences will be checked and verified using the “Submission Policy” (see http://statdb.bic.nus.edu.sg/downloads/submission_policy.pdf). This would result in a database rich with annotations by expert curators in the field.

**iv. Classification**

STATs are complex proteins, but have been originally classified based simply on function and named according to their order of discovery (STAT types) [1], [12], [13]. The mammalian STAT family comprises seven different known members (STAT1-4, STAT5A, STAT5B and STAT6), which correspond to a determined function (see Table W4 at “STATdb Home > Classification”), and other types commonly found in fishes or invertebrates. The “STAT (s)” annotation is used to refer to the family or species-specific STATs, and the “(s)” represents the literature name of the STAT in the particular species. This includes STAT (dstA to D) of *Dictyostelium discoideum* and *Polysphondylium pallidum PN500*, STAT (D-STAT) of *Drosophila melanogaster*, and STAT (STA-1) and STAT (STATB) of *C. elegans*. Unknown, predicted or hypothetical STATs are denoted as STAT(u).

Although sequences of a STAT type are described to share the same function, our analysis shows that they possess differences in their domain architecture and, in some cases, appear to be lineage-specific [10], [31], [32], [33], [34]. As such, there might be subtle but distinct differences in the mode of function between family members of a STAT type, which merits further investigation. The rationale behind our classification system was to further stratify the original classification in a way that would allow for the quick delineation of possible structure, function and lineage of novel STATs.

The analysis of STAT type by structure revealed five distinct domain architectures (see Table W2 at “STATdb Home > Classification”). Domain architecture I (DA I) contains all of the five domains in the order of STAT_int, STAT_alpha, STAT_bind, SH2 and TAZ2 from N- to C-terminus (see Materials and Methods for their descriptions). DA II lacks the TAZ2 domain, whereas DA III lacks both the TAZ2 and the STAT_int. DA IV contains only the STAT_bind and SH2 domains, and DA V comprises the coiled-coil domain (Dict_STAT_coil) and the SH2 domain. All other sequences that cannot be classified in this way—but contain or show similarity to at least one of the five major domains—are labeled as DA U. All artificial sequences, even if they share the observed orders, are still classified as DA U.

The five domain architectures can be further differentiated into 14 unique lineages, notated as “A” to “M”, with “Z” for artificial sequences (see Table W3 at “STATdb Home > Classification”). Two lineages were observed for each bilateria (Deuterostomia and Protostomia), cnidaria (Anthozoa and Hydrozoa), choanoflagellida (Monosiga and Salpingoeca), and dictyosteliida (Dictyostelium and Polysphondylium), whereas one lineage was observed for each placozoa (trichoplax), porifera (demospongiae), ichthyosporea (Capsaspora), acanthamoeba and tracheophyta.

The stratification of STAT types into “DA” and “Lineage” resulted in a three-tier classification system, with notations, such as “DA I : A : STAT1”, which describes “DA” (tier one), “Lineage” (tier two), and “Function” (tier three), respectively (Figure 2C). Currently, the website comprises 96 notations that involve three tiers (see Table W1 at “STATdb Home > Classification”). Searches can thus be performed according to these collective notations or to each individual tier; this information is provided under the “Classification” section (“STATdb Home > Classification”) or can be found using the “Search” page (“STATdb Home > Search”). The classification will be updated regularly to provide a true representation of the STATome as the database grows.

**v. Predict**

**Figure 3:**
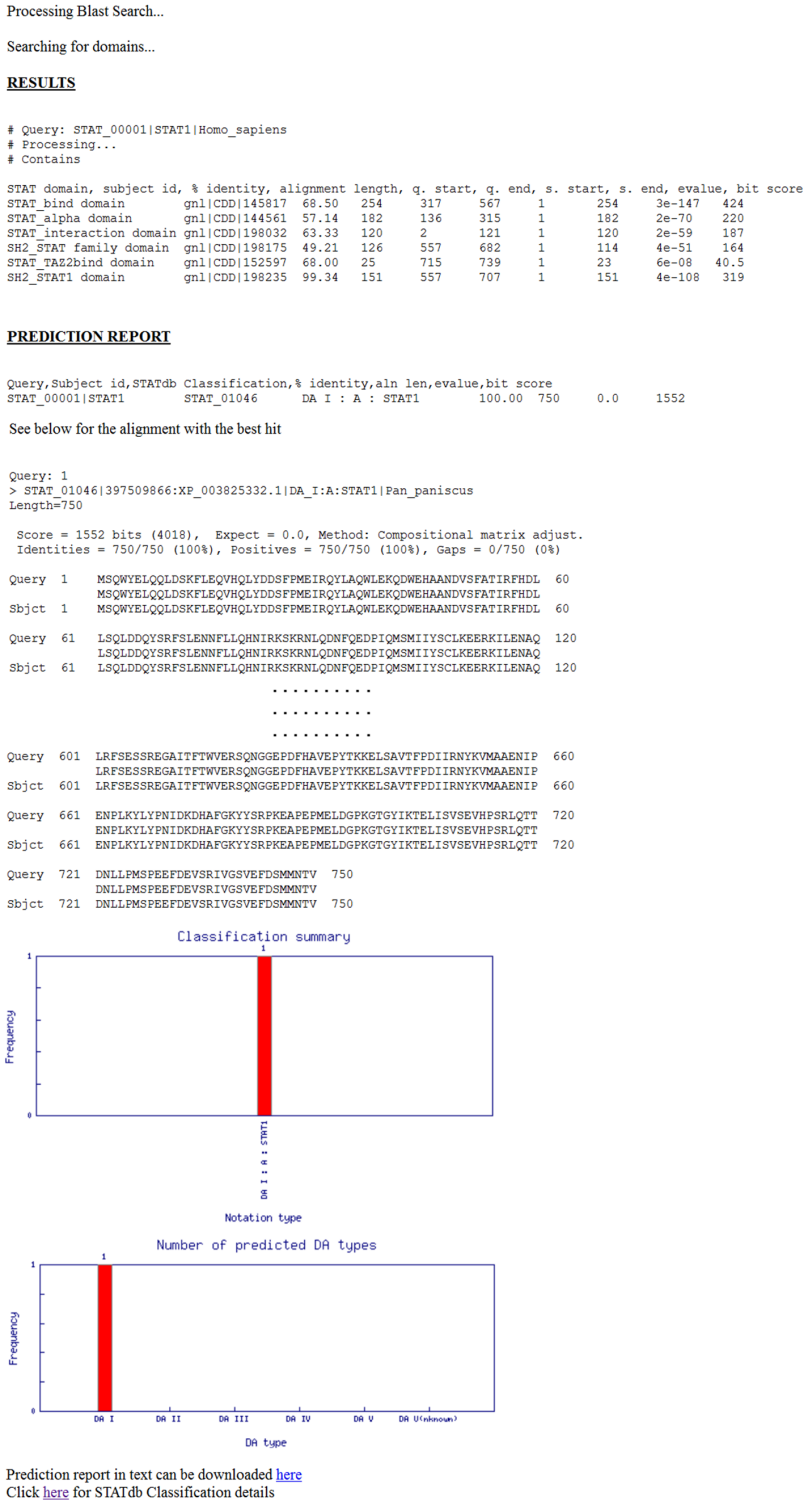
STATdbPredict output report page for STAT_00001. The alignment is cropped to save space.

STATdbPredict characterizes protein sequences through the STATdb classification system (Figure 2D). This prediction system reports the STATdb classification notation of the query sequence(s) along with any additional information, such as individual domain hits, the HSP, and the frequency of the different notations (Figure 3). This provides information on the potential structure, function and lineage of novel STATs, which can help in planning experiments for validation. STATdbPredict is essentially a combination of two BLAST programs: RPS-BLAST (against an in-house database of PSSM matrices downloaded from CDD) and standard BLASTp (against STATdb version without the test dataset) with optimized parameters that are applied in the context of the 3-tier classification. Outputs of STATdbPredict are annotated according to the classification, whereas a standalone BLAST search against STATdb sequences also annotated according to the 3-tier annotation would provide a similar result but of lower overall accuracy (∼91% versus ∼94%) and sensitivity (∼82% versus ∼89%) than STATdbPredict (Table 2). This is because RPS-BLAST, through the use of PSSM matrices, captures the diversity of the domains, which cannot be represented by a single HSP of a BLAST search. Even though the percentage differences in accuracy between Predict and standalone BLAST seem minor, the absolute number of records affected is significant and will be more so for a larger data size; for example, 17 were incorrectly identified by standalone BLAST for a test dataset of 96 positive and 96 negative samples. Nonetheless, both methods have a high overall accuracy because of the granular stratification of STAT sequences into the 3-tier classification system. The prediction system will be updated regularly for improved reliability as the size of the database grows. STATdb represents a platform for the future development of more sophisticated meta-predictors, with an increased number of record and corresponding annotations for scanning the tree of life genome/proteome for novel STATs in practical applications.

**Table 2:** Performance measures of STATdbPredict (RPS-BLAST and BLASTp) versus standalone BLASTp search.

A. STATdbPredict search							
	TP	FN	TN	FP	Accuracy (%)	Sensitivity (%)	Specificity (%)
**Domain Architecture (DA)**	85	11	96	0	94.27	88.54	100
**Lineage**	95	1	96	0	99.48	98.96	100
**STAT type**	90	6	96	0	96.88	93.75	100

B. Standalone BLASTp search							
	TP	FN	TN	FP	Accuracy (%)	Sensitivity (%)	Specificity (%)
**Domain Architecture (DA)**	79	17	96	0	91.15	82.29	100
**Lineage**	95	1	96	0	99.48	98.96	100
**STAT type**	90	6	96	0	96.88	93.75	100

### Application of STATdbPredict: Defining the STATome

The STATome represents all reported STAT sequences in nature. The sequences used to populate STATdb were obtained via two approaches: (i) a standard search of NCBI NR (see Materials and Methods for “Sequence Data Collection”) and (ii) STATdbPredict to scan UniProt UniRef100 [35] and NCBI NR datasets. At the time of collection, the UniRef100 dataset contained 20,002,214 sequences, whereas the NR dataset contained 23,075,327 sequences. The standard search returned 1,126 STAT sequences, whereas STATdbPredict identified an additional 65 unique sequences from NR and 233 from UniRef100. In addition, the 116 sequences identified during the accuracy analysis of STATdbPredict, which were obtained more recently using a standard NCBI NR search, were eventually included in STATdb, resulting in a total of 1,540 distinct sequence records.

STATdb is currently the only specialised repository of the STATome. Of the 1,540 records (as at June 2013), 186 are experimentally (“E”) verified STAT sequences, whereas 1,354 are predicted (“P”) (see submission/curation policy for grouping procedure). A total of 93 records have annotations of the interacting partners, which broadly fall under four groups: inhibitors, such as protein inhibitor of activated STAT (PIAS), and suppressors of cytokine signaling (SOCS); activators, such as JAK, Src kinase and EGFR; cytokines, such as interferons and interleukins, which comprise the majority; and unclassified, such as JUN, BCL3, Gfap, EP300, among others. STAT is currently reported to be present in 235 species from diverse lineages, including bilateria, cnidaria, choanoflagellida, dictyosteliida, placozoa, porifera, ichthyosporea, acanthamoeba and tracheophyta. The STAT types—STAT1, STAT2, STAT4, STAT5A, STAT5B and STAT6—are represented by more than 100 records each, whereas STAT3 and STAT(u) comprise over 200 and 300 records, respectively (navigate to “STATdb Home > Help > Statistics” for the list of species and STAT types).

### Maintenance, stability and growth of STATdb

We have devised an update policy (see http://statdb.bic.nus.edu.sg/downloads/update_policy.pdf) for the regular growth of the database. The stability of the database will be monitored regularly, and feedback from users will be key in addressing any bugs or issues within the system. Additionally, regression testing will be performed before major updates to ensure full functionality and stability. Plans for longevity of the database beyond the current team include a proposal for the long-term maintenance of the database by a group of volunteers selected from the list of top contributors. These users will be given the authority to make changes to the database in accordance with the standard system administrator acceptable use policy, and will also be responsible for maintaining the various policies of the database, such as new sequence submissions and update policies. Other plans include depositing the latest copy to Asia-Pacific Bioinformatics Network's (APBioNet's) cloud re-instantiation Web-accessible system (http://biodb100.apbionet.org; [36]) for archival and future on-demand re-instantiation by users where the original database site is not accessible. This is in line with the Minimum Information about a Bioinformatics Investigation (MIABi) standards [37], harmonised with the BioDBcore standards of the International Society for Biocuration (ISB) and BioSharing [38].

## Discussion

STATdb is a unique Web resource that provides a comprehensive collection of STAT protein and nucleotide sequences, enriched with functional and structural annotations for data mining and analyses. The significant attributes of STATdb include: (a) integration of STAT data from different databases, creating the only unified STATome reported to date; (b) a novel classification system (comprising characteristic features of STAT protein sequences), which is used as a basis for STATdbPredict, a high accuracy (>90%) meta-predictor; (c) tools to analyse the functional and structural properties of STAT (BLAST, alignment, STATdbPredict); and (d) a platform for community contribution, which is guided by submission curation and an update policy. We envisage that this database will serve as a template for the development of a knowledgebase for signaling proteins.
